# IGF2BP2 Promotes Esophageal Squamous-Cell Carcinoma Progression with Potential Involvement of PI3K/AKT Signaling

**DOI:** 10.3390/cimb48070726

**Published:** 2026-07-16

**Authors:** Xiaohang Gao, Minghui Yang, Jing Yang, Yunpeng Zhong, Chao Ma, Guoliang Xu, Lin Zhang, Rong Zhang

**Affiliations:** 1 Department of Radiation Oncology, State Key Laboratory of Oncology in South China, Guangdong Provincial Clinical Research Center for Cancer, Sun Yat-sen University Cancer Center, Guangzhou 510060, China; gaoxh1@sysucc.org.cn; 2 Department of Endoscopy, State Key Laboratory of Oncology in South China, Guangdong Provincial Clinical Research Center for Cancer, Sun Yat-sen University Cancer Center, Guangzhou 510060, China; yangmh@sysucc.org.cn (M.Y.); zhongyunpeng@sysucc.org.cn (Y.Z.); xugl@sysucc.org.cn (G.X.); 3 Department of Internal Medicine, State Key Laboratory of Oncology in South China, Guangdong Provincial Clinical Research Center for Cancer, Sun Yat-sen University Cancer Center, Guangzhou 510060, China; yangjing2@sysucc.org.cn; 4 Department of Pathology, State Key Laboratory of Oncology in South China, Guangdong Provincial Clinical Research Center for Cancer, Sun Yat-sen University Cancer Center, Guangzhou 510060, China; machao@sysucc.org.cn; 5 Department of Clinical Laboratory, State Key Laboratory of Oncology in South China, Guangdong Provincial Clinical Research Center for Cancer, Sun Yat-sen University Cancer Center, Guangzhou 510060, China

**Keywords:** IGF2BP2, esophageal squamous cell carcinoma (ESCC), PI3K/AKT-related signaling, cell proliferation, EMT-associated markers, candidate biomarker

## Abstract

Esophageal squamous-cell carcinoma (ESCC) remains a highly lethal malignancy, with long-term survival still unsatisfactory and an urgent need for clinically relevant molecular targets. Insulin-like growth factor 2 mRNA-binding protein 2 (IGF2BP2) is an RNA-binding protein with oncogenic activity in several tumor types, but the signaling events associated with its role in ESCC have not been fully defined. IGF2BP2 expression was examined in paired ESCC and adjacent non-tumor tissues by immunohistochemistry (10 paired samples) and qRT-PCR (16 paired samples). Associations with clinicopathological variables were evaluated in 108 TCGA ESCC cases. Stable lentiviral shRNA-mediated knockdown was established in KYSE30 and ECA109 cells. CCK-8, colony formation, Transwell, EdU, and flow cytometry assays were used to assess proliferation, clonogenic growth, migration, invasion, apoptosis, and cell-cycle distribution. EMT-associated markers were measured by Western blotting and qRT-PCR. RNA sequencing, KEGG enrichment analysis, and rescue experiments using the AKT activator SC79 were performed to explore downstream signaling. IGF2BP2 was upregulated in ESCC tissues at both the transcript and protein levels, and higher expression was associated with unfavorable clinicopathological characteristics. Silencing IGF2BP2 reduced proliferation, colony formation, migration, and invasion, promoted G0/G1 cell-cycle arrest, and increased apoptosis. EMT-associated marker analysis showed decreased Snail and increased Slug expression, indicating that these changes require cautious interpretation. RNA sequencing and KEGG analysis suggested enrichment of PI3K/AKT-related signaling after IGF2BP2 knockdown. IGF2BP2 silencing decreased PI3K and p-PI3K levels, whereas SC79 partly restored migratory and invasive capacities. IGF2BP2 contributes to malignant phenotypes in ESCC and may be associated with PI3K/AKT-related signaling. These findings indicate that IGF2BP2 is a candidate biomarker and potential therapeutic target, although further mechanistic and pharmacologic validation is required.

## 1. Introduction

Esophageal cancer is a major global health burden and remains among the leading causes of cancer-related death worldwide [1,2]. The two major histological subtypes are esophageal adenocarcinoma (EAC) and esophageal squamous-cell carcinoma (ESCC). ESCC represents the dominant subtype worldwide and accounts for the great majority of esophageal cancer cases in China [3,4,5,6]. This malignancy originates from the squamous epithelium of the esophagus and usually displays aggressive clinical behavior. Because many patients are diagnosed only after the disease has reached a locally advanced or metastatic stage, prognosis remains poor. For advanced disease, median survival is commonly less than 12 months, and the overall 5-year survival rate remains below 20% [7]. Although systemic chemotherapy combined with immunotherapy has improved treatment options for advanced esophageal cancer, the molecular drivers of ESCC progression are still not fully understood. Defining these mechanisms may therefore help identify more effective therapeutic strategies and improve patient outcomes.

IGF2BP2 is a member of the insulin-like growth factor 2 mRNA-binding protein family (IGF2BPs). These conserved RNA-binding proteins contain two N-terminal RNA recognition motifs and four C-terminal K-homology domains, which enable regulation of mRNA localization, translation, and stability [8,9]. IGF2BP2 has been associated with diverse biological and pathological processes, including metabolic regulation, angiogenesis, immune modulation, embryonic development, and epigenetic regulation [10,11]. IGF2BP family proteins, including IGF2BP2, can function as readers of N6-Methyladenosine (m6A), a common internal modification in eukaryotic mRNA involved in cell development, differentiation, and tumorigenesis [12,13]. Increased IGF2BP2 expression has been observed in multiple cancers, including ovarian, breast, lung, cervical, melanoma, colorectal, leukemia, and head and neck malignancies, where it has been linked to proliferation, migration, invasion, and tumor growth [14,15,16,17,18,19,20,21]. These observations suggest that IGF2BP2 may act as a tumor-promoting RNA-binding protein.

The role of IGF2BP2 in ESCC has been explored in previous studies, but the signaling pathways related to its oncogenic function require further clarification. Reported mechanisms include enhanced EIF4A1 translation, stabilization of OCT4 mRNA, and stabilization of HDGF mRNA, all of which support ESCC-associated malignant phenotypes [22,23,24]. These findings indicate that IGF2BP2 can regulate ESCC progression through different downstream RNA targets; however, the broader signaling context through which IGF2BP2 influences ESCC progression remains incompletely defined.

Here, we evaluated the relationship between IGF2BP2 expression and clinicopathological characteristics in ESCC and examined the functional consequences of IGF2BP2 knockdown in ESCC cells. Transcriptomic profiling was used to identify genes and pathways altered by IGF2BP2 silencing, followed by functional experiments to explore possible downstream mechanisms. This work provides additional evidence supporting the tumor-promoting role of IGF2BP2 in ESCC.

## 2. Material and Methods

### 2.1. Cell Culture

KYSE30, KYSE150, KYSE410, TE-1, and ECA109 human ESCC cell lines, together with the normal esophageal epithelial cell line HET-1A, were obtained from the Cell Research Institute of the Chinese Academy of Sciences. Cells were maintained in RPMI-1640 medium (C11995500BT, Gibco, Thermo Fisher Scientific, Waltham, MA, USA) containing 10% fetal bovine serum (164210-50, Gibco) and 1% penicillin–streptomycin (GX15140, Gingxin, Shanghai, China) at 37 °C in a humidified 5% CO_2_ incubator.

### 2.2. Patients and Clinical Tumor Samples

ESCC tumor tissues and matched adjacent normal tissues were collected from newly diagnosed patients undergoing diagnostic endoscopic biopsy at Sun Yat-sen University Cancer Center in 2024. Only residual endoscopic biopsy material obtained during routine pathological diagnosis was used. Ten paired tumor and adjacent normal specimens were analyzed by immunohistochemistry, an independent cohort of 16 paired specimens was used for qRT-PCR validation, and a separate set of 10 paired specimens was used for tissue RNA sequencing. The immunohistochemistry, qRT-PCR, and tissue RNA-seq cohorts were independent and did not overlap. Clinicopathological information was available for the 16-patient qRT-PCR cohort and is summarized in Supplementary Table S3. Patient-level data were not retained for the immunohistochemistry or tissue RNA-seq specimens because the samples had been deidentified before experimental analysis.

The study protocol was approved by the Ethics Committee of Sun Yat-sen University Cancer Center (approval No. SZR2021-124). Individual informed consent was waived because this was a retrospective study using residual clinical specimens and deidentified clinicopathological information. All procedures were conducted in accordance with the Declaration of Helsinki and institutional requirements. None of the included patients had received chemotherapy or radiotherapy before specimen collection. After routine pathological evaluation, residual deidentified tissue was transferred to the laboratory for research use.

### 2.3. Lentiviral Transfection

pPLK/GFP + Puro lentiviral constructs carrying IGF2BP2 shRNA were designed and synthesized by the Public Protein/Plasmid Library (Nanjing, China). ESCC cells were infected with lentiviral vectors targeting IGF2BP2 or with a negative-control shRNA vector. The control group was named sh-NC, and the knockdown groups were named shIGF2BP2-1, shIGF2BP2-2, and shIGF2BP2-3. Stable cell lines were generated after 2 weeks of puromycin selection (1.5 μg/mL, ST551, Beyotime Biotechnology, Shanghai, China). Knockdown efficiency was evaluated 48 h after transfection using qPCR and Western blotting. Primer sequences are provided in Supplementary Table S1.

### 2.4. Cell Counting Kit-8 Assay

After lentiviral transfection, KYSE30 and ECA109 cells were plated in 96-well plates at 2000 cells per well and cultured for 72 h. The medium was discarded, and each well received 10 μL CCK-8 reagent (C0038, Beyotime, Shanghai, China) plus 100 μL serum-free medium under light-protected conditions. Following a 2-h incubation at 37 °C, absorbance was read at 450 nm with a microplate reader.

### 2.5. TCGA Data Analysis

GEPIA was used to perform pan-cancer expression analysis and to compare IGF2BP2 expression between esophageal carcinoma and normal tissues using TCGA and GTEx data. For clinicopathological correlation analysis, 108 TCGA ESCC cases with available annotations were divided into high- and low-expression groups according to the median IGF2BP2 level. Because the GEPIA expression comparison was based on the broader TCGA-ESCA dataset, the database findings were considered supportive and interpreted together with the institutional ESCC validation cohorts.

### 2.6. Immunohistochemistry

Paraffin-embedded sections were heated, deparaffinized with xylene, and rehydrated using a graded ethanol series. After washing three times with PBS, endogenous peroxidase activity was blocked with 3% H_2_O_2_ for 10 min, followed by additional PBS washes. Sections were incubated with 10% serum blocking solution for 30 min and then with the primary antibody overnight at 4 °C. On the following day, the slides were washed twice with PBS, incubated with biotin-labeled secondary antibodies for 1 h in the dark, developed with DAB, and counterstained with hematoxylin for 2.5 min until uniform nuclear staining was observed. The staining reaction was stopped by rinsing with tap water (Shunning, Ningbo Shunning Instruments Co., Ltd., Ningbo, China).

### 2.7. Colony Formation

KYSE30 and ECA109 cells were plated in 12-well plates at 450 cells per well, with three replicate wells for each condition. Cells were cultured for 2 weeks, and the medium was refreshed every 3 days. Colonies were fixed with 0.5 mL 4% paraformaldehyde for 20 min after PBS washing and then stained with 1 mL 0.5% crystal violet (Beyotime, Shanghai, China). After washing and air drying, colonies containing more than 50 cells were counted microscopically (Shunning, Ningbo Shunning Instruments Co., Ltd., Ningbo, China).

### 2.8. EdU Experiment

For EdU assays, ESCC cells were seeded in 12-well plates at 500 cells per well. EdU reagent was added, and cells were incubated for 2 h before fixation with 4% paraformaldehyde. Cells were washed three times with detergent solution containing 0.3% BSA and permeabilized with 0.3% Triton X-100 in PBS for 15 min at room temperature. According to the EdU Proliferation Kit protocol (C0071S, Beyotime, Shanghai, China), cells were incubated with 0.5 mL Click reaction solution for 30 min in the dark, followed by staining with 1× Hoechst33342 for 10 min. Fluorescence images were acquired using a fluorescence microscope (Shunning, Ningbo, China).

### 2.9. Transwell Assay

For Transwell assays, 200 μL of ESCC cell suspension (5 × 10^5^ cells per well) was added to the upper chamber of a 24-well Transwell insert, and 600 μL of medium containing 15% FBS was added to the lower chamber. For migration assays, uncoated Transwell inserts were used. For invasion assays, the upper chambers were pre-coated with Matrigel according to the manufacturer’s instructions before cell seeding. Where indicated, SC79 (4 μg/mL; HY-18749-5, MedChemExpress, Monmouth Junction, NJ, USA) was added to the upper chamber. The cells were incubated for 24 h. The inserts were subsequently washed with PBS, fixed with 4% paraformaldehyde for 10 min, and stained with 0.5% crystal violet for 10 min. After washing and air drying, cells that had migrated or invaded through the membrane were counted under a microscope.

### 2.10. Cell-Cycle Assay

For cell-cycle analysis, ESCC cells were seeded in 6-well plates at 3 × 10^5^ cells per well and cultured for 24 h. Cells were detached with trypsin (25200072, Gibco, Thermo Fisher Scientific, Waltham, MA, USA), resuspended in PBS, and slowly added to 1 mL 75% ethanol for overnight fixation at 4 °C. After centrifugation and PBS washing the next day, cells were treated with 1 mL RNase and 200 μL PI staining solution. Samples were incubated for 30 min at room temperature in the dark, analyzed on a Novocyte D2060R flow cytometer(Agilent Technologies, Santa Clara, CA, USA), and processed with FlowJo software (v10.8, FlowJo LLC, Ashland, OR, USA).

### 2.11. RNA Extraction and qPCR

Total RNA from cells or tissues was isolated using RNAiso Plus (9109, Takara Bio Inc., Kusatsu, Shiga, Japan), and RNA concentration was determined with a spectrophotometer. cDNA was generated using the Hifair II 1st Strand cDNA Synthesis Kit with gDNA Eraser (11119ES60, Yeasen Biotechnology (Shanghai) Co., Ltd., Shanghai, China) following the manufacturer’s protocol. qPCR reactions were prepared and run on a qPCR system. The primer sequences used for qRT-PCR are provided in Supplementary Table S2. Relative gene expression was quantified using the 2^−ΔΔCt^ method.

### 2.12. Western Blot Analysis

Cells were lysed in RIPA buffer (P0013, Beyotime Biotechnology, Shanghai, China) containing 1% phosphatase inhibitor and 1% PMSF protease inhibitor. Lysates were kept on ice for 25 min and centrifuged, and the resulting supernatants were stored at −80 °C. Protein concentrations were measured using a BCA protein assay kit (BL521A, Biosharp Life Sciences, Hefei, China) at 562 nm. Equal protein amounts were resolved by SDS-PAGE and transferred to PVDF membranes. After blocking with 5% skim milk for 2 h, membranes were incubated overnight at 4 °C with primary antibodies against IGF2BP2, PI3K, p-PI3K, AKT, p-AKT, E-cadherin, Vimentin, Snail, Slug, and GAPDH. The IGF2BP2 antibody was from Proteintech (11601-1-AP, Rosemont, IL, USA). Detailed primary antibody information, including target, vendor, catalog number, dilution, and incubation conditions, is listed in Supplementary Table S4. After three washes with Tris-buffered saline with Tween (TBST), membranes were incubated with secondary antibodies for 2 h at room temperature. Equal volumes of ECL solutions A and B (BL520B, Biosharp) were mixed and applied to the membranes, and chemiluminescence was detected using a Saizhi imaging system (Beijing, China).

### 2.13. RNA Sequencing and Bioinformatics Analysis

For tissue RNA-seq, total RNA was isolated from 10 paired ESCC tumor and adjacent normal specimens. For knockdown RNA-seq, total RNA was isolated from ECA109 cells stably expressing shIGF2BP2-1 or sh-NC, with three biological replicates per group (sh-NC: NC1, NC2, and NC3; shIGF2BP2-1: SH1, SH2, and SH3). RNA concentration, purity, and integrity were assessed before library construction. Sequencing libraries were prepared according to the manufacturer’s protocol and sequenced using the DNBSEQ-T7 platform (MGI Tech Co., Ltd., Shenzhen, China). Raw reads were processed to remove adapter sequences, reads with more than 10% ambiguous bases, and reads with more than 50% low-quality bases (Q ≤ 5). Clean reads were aligned to the human GRCh38/hg38 reference genome, and gene-level count and normalized expression matrices were produced for downstream analyses. Sequencing yield and quality metrics, including raw reads, clean reads, Q20, Q30, and GC content, were documented in the quality-control report and GEO submission files.

Differential expression analysis was conducted for ESCC tumor versus adjacent normal tissues and for IGF2BP2-knockdown versus sh-NC ECA109 cells. Genes with a Benjamini–Hochberg adjusted *p* value < 0.05 and an absolute log2 fold change > 1 were considered differentially expressed. GO analysis was used to characterize associated biological processes, whereas KEGG analysis was used to identify enriched pathways. Bioinformatic processing and visualization were performed using the provider’s Bulk RNA-seq analysis platform together with R software (4.6.1; R Foundation for Statistical Computing, Vienna, Austria).

### 2.14. Statistical Analysis

Statistical analyses were carried out using SPSS version 27.0.1. Quantitative data were derived from at least three independent experiments and are shown as mean ± standard deviation (SD). Student’s *t*-test was used for two-group comparisons, and one-way analysis of variance (ANOVA) was used for comparisons involving more than two groups. Pearson’s correlation analysis was applied to examine the relationship between IGF2BP2 expression and pathway-related biomarkers. A two-sided *p* value < 0.05 was considered statistically significant.

## 3. Results

### 3.1. Expression Level of IGF2BP2 in Esophageal Squamous-Cell Carcinoma Samples

**Figure 1:**
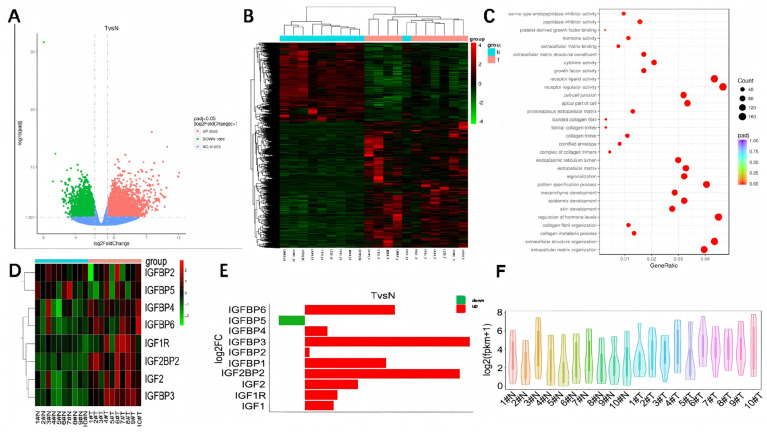
IGF2BP2 is markedly upregulated in esophageal squamous-cell carcinoma tissues based on transcriptomic profiling. (**A**) Volcano plot illustrating differentially expressed genes identified by RNA sequencing in 10 paired ESCC tumor (T) versus adjacent normal (N) tissue samples. Genes meeting the criteria of adjusted *p* value < 0.05 and |log2 fold change| > 1 were considered differentially expressed, comprising 3035 upregulated (red) and 1886 downregulated (green) genes. IGF2BP2 is highlighted among the significantly upregulated genes. (**B**) Hierarchical clustering heatmap displaying the expression patterns of differentially expressed genes across all paired ESCC tumor (T) and adjacent normal (N) tissue samples. (**C**) Gene Ontology enrichment analysis of differentially expressed genes between ESCC and adjacent normal tissues. Selected enriched GO terms generated by GO analysis are shown and presented according to the original enrichment output. The x-axis represents the GeneRatio, calculated as the proportion of differentially expressed genes annotated to each GO term. Dot size represents the number of genes enriched in each term, and dot color indicates the adjusted *p* value. ESCC, esophageal squamous-cell carcinoma; GO, Gene Ontology. (**D**) Heatmap illustrating the expression levels of IGF family members across paired ESCC tumor and adjacent normal tissues, revealing upregulation of most members except IGFBP5. (**E**) Bar chart depicting log2-fold changes in IGF family members in ESCC tumor relative to adjacent normal tissues (TvsN). IGF2BP2 was among the most substantially upregulated members within this family. (**F**) Violin plots showing the distribution of IGF2BP2 expression [log2(FPKM + 1)] in 10 paired adjacent normal (N) and ESCC tumor (T) specimens, confirming consistently higher expression levels in tumor tissues.

Although IGF2BP2 overexpression in ESCC has been described previously, we performed RNA-seq to define the transcriptomic profile of our institutional ESCC specimens and to select dysregulated genes for functional analysis. In 10 paired tumor and adjacent normal tissue samples, 3035 genes were upregulated and 1886 genes were downregulated in tumor tissues (Figure 1A,B). GO analysis indicated that these differentially expressed genes were enriched mainly in receptor- and ligand-associated biological processes, providing a transcriptomic basis for identifying candidate genes such as IGF2BP2 (Figure 1C). IGF2BP2 was markedly increased among the upregulated genes and was therefore selected for validation and mechanistic study (Figure 1D). Analysis of IGF family genes showed broad upregulation of most members except IGFBP5, with IGF2BP2 among the more highly expressed genes in ESCC samples (Figure 1E). A violin plot further showed consistently higher IGF2BP2 expression in tumor tissues (Figure 1F).

### 3.2. Expression Level of IGF2BP2 in TCGA and Institutional Validation Cohorts

**Figure 2:**
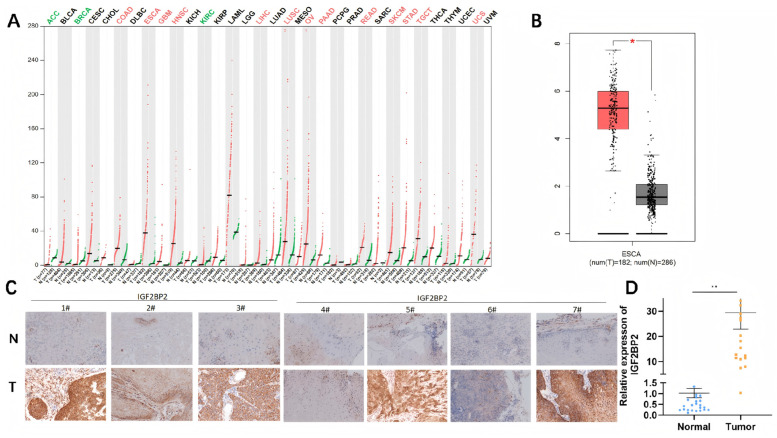
IGF2BP2 expression in public datasets and institutional ESCC validation cohorts. (**A**) GEPIA-based pan-cancer analysis showing IGF2BP2 expression across tumor types in TCGA. Red indicates tumor types with higher expression relative to normal tissues, and green indicates lower expression. (**B**) Box plot comparing IGF2BP2 mRNA expression between esophageal carcinoma tissues (T, *n* = 182) and normal tissues (N, *n* = 286) in the TCGA-ESCA/GTEx dataset. The red box represents tumor tissues, and the gray box represents normal tissues (* *p* < 0.05). (**C**) Representative IHC images from paired ESCC tumor and adjacent normal tissues, showing stronger cytoplasmic IGF2BP2 staining in tumor specimens (magnification: 100×). (**D**) qRT-PCR analysis of 16 paired ESCC tumor and adjacent normal specimens, showing significantly higher IGF2BP2 expression in tumor tissues (** *p* < 0.01). Blue circles indicate adjacent normal tissues, whereas orange squares indicate ESCC tumor tissues.

To assess the clinical relevance of IGF2BP2, we analyzed public TCGA data using GEPIA and an ESCC-specific TCGA clinicopathological cohort. Pan-cancer analysis indicated that IGF2BP2 was elevated in several tumor types, including colorectal cancer, glioblastoma, head and neck squamous-cell carcinoma, lung cancer, and ovarian cancer (Figure 2A). GEPIA analysis further showed higher IGF2BP2 expression in esophageal carcinoma tissues than in normal tissues (Figure 2B). In our institutional cohort, IHC using 10 paired specimens confirmed increased IGF2BP2 protein expression in ESCC tissues (Figure 2C), and qRT-PCR analysis of 16 paired specimens showed higher IGF2BP2 mRNA expression in tumors (Figure 2D). In the TCGA ESCC cohort, increased IGF2BP2 expression was associated with age (*p* = 0.030), smoking history category (*p* = 0.006), lymph node metastasis (*p* = 0.031), and histological grade (*p* = 0.014) (Table 1). These results support an association between IGF2BP2 upregulation and ESCC progression.

### 3.3. IGF2BP2 Promotes ESCC Cell Proliferation

**Figure 3:**
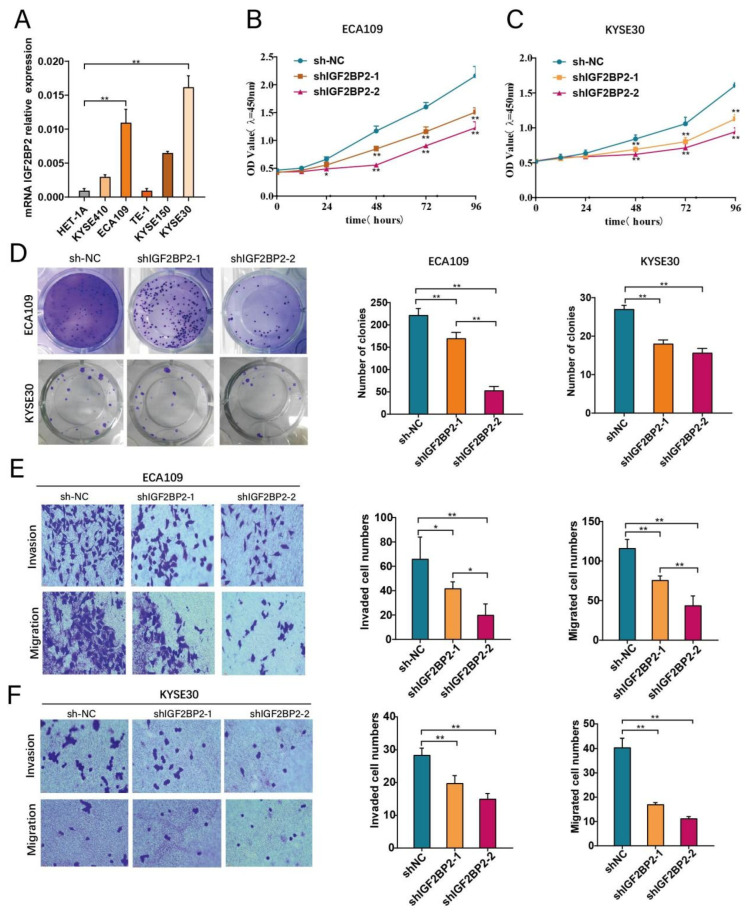
Effects of IGF2BP2 knockdown on ESCC cell growth and motility. (**A**) qRT-PCR analysis of IGF2BP2 mRNA levels in five ESCC cell lines and HET-1A cells; KYSE30 and ECA109 cells showed relatively high endogenous expression and were used for subsequent assays (** *p* < 0.01). (**B**,**C**) CCK-8 assays in ECA109 and KYSE30 cells expressing sh-NC, shIGF2BP2-1, or shIGF2BP2-2 over 96 h. IGF2BP2 knockdown decreased cell viability compared with sh-NC (* *p* < 0.05, ** *p* < 0.01). (**D**) Representative colony formation images and quantification showing fewer colonies after IGF2BP2 knockdown. (**E**,**F**) Representative Transwell images and quantification of invasion and migration in ECA109 and KYSE30 cells, showing reduced invasive and migratory capacity after IGF2BP2 depletion (* *p* < 0.05, ** *p* < 0.01). Data are shown as mean ± SD from three independent experiments.

We then evaluated IGF2BP2 expression in ESCC cell models. qPCR showed that ECA109, KYSE410, KYSE30, KYSE150, and TE-1 cells expressed higher IGF2BP2 levels than HET-1A normal esophageal epithelial cells (Figure 3A). Available demographic and clinicopathological characteristics of the institutional validation cohort are provided in Supplementary Table S3. Because KYSE30 and ECA109 cells showed relatively high endogenous IGF2BP2 expression, they were selected for subsequent functional assays. Stable shRNA-mediated knockdown was established, and reduced IGF2BP2 expression was confirmed by qPCR and Western blotting (Figure S1A).

Functional assays indicated that reducing IGF2BP2 expression weakened malignant phenotypes in ESCC cells. CCK-8 assays showed decreased proliferation after knockdown (Figure 3B,C), and colony formation assays demonstrated reduced clonogenic growth (Figure 3D). EdU staining also revealed lower proliferative activity after IGF2BP2 silencing (Figure S2). Transwell assays further showed that knockdown of IGF2BP2 decreased migration and invasion (Figure 3E,F). Overall, these findings indicate that IGF2BP2 supports ESCC cell proliferation, migration, and invasion.

### 3.4. Effects of IGF2BP2 on Apoptosis and Cell-Cycle Progression

**Figure 4:**
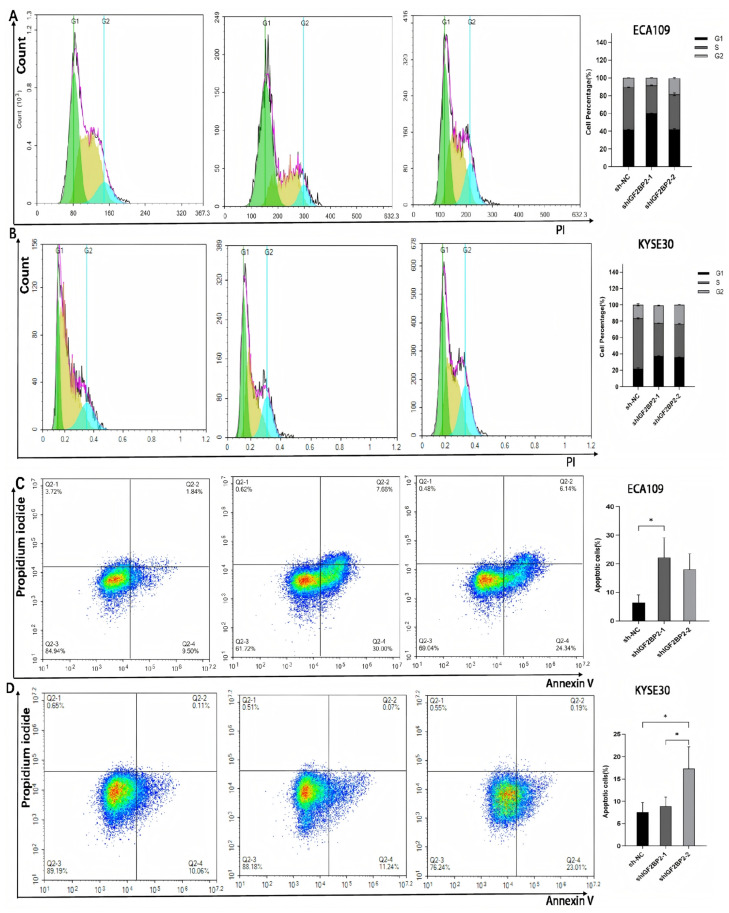
IGF2BP2 depletion affects cell-cycle progression and apoptosis in ESCC cells. (**A**,**B**) Cell-cycle profiles of ECA109 and KYSE30 cells after IGF2BP2 knockdown. Representative PI-stained flow cytometry plots and quantification of G0/G1, S, and G2/M phase distributions are shown for sh-NC, shIGF2BP2-1, and shIGF2BP2-2 groups. In the cell-cycle profiles, the green, yellow, and blue areas represent the G0/G1, S, and G2/M phases, respectively. In the stacked bar graphs, black, dark-gray, and light-gray segments represent the G0/G1, S, and G2/M phases, respectively. (**C**,**D**) Representative Annexin V-FITC/PI apoptosis plots and quantification of early and late apoptotic cells in ECA109 and KYSE30 cells. In the pseudocolor plots, warmer colors indicate higher event density and cooler colors indicate lower event density; the lower-right and upper-right quadrants represent early and late apoptotic cells, respectively. In the quantification graphs, black, dark-gray, and light-gray bars represent the sh-NC, shIGF2BP2-1, and shIGF2BP2-2 groups, respectively.Apoptosis increased in shIGF2BP2 groups compared with sh-NC controls (* *p* < 0.05). Data are presented as mean ± SD from three independent experiments.

We next examined whether IGF2BP2 affected cell-cycle distribution and apoptosis. Flow cytometry showed that IGF2BP2 knockdown increased the G0/G1-phase population and reduced S-phase entry in ECA109 and KYSE30 cells (Figure 4A,B). IGF2BP2 silencing also increased apoptosis compared with the control group (Figure 4C,D). These data suggest that IGF2BP2 may promote ESCC cell growth by facilitating G1/S transition and limiting apoptosis.

**Figure 5:**
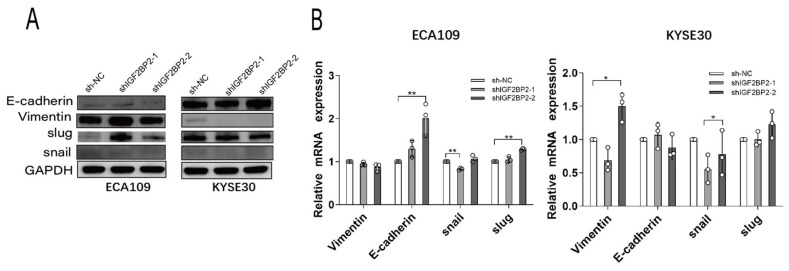
IGF2BP2 knockdown changes EMT-associated marker expression in ESCC cells. (**A**) Western blotting of E-cadherin, Vimentin, Slug, and Snail in ECA109 and KYSE30 cells expressing sh-NC, shIGF2BP2-1, or shIGF2BP2-2. GAPDH was used as the loading control. IGF2BP2 depletion was associated with increased E-cadherin, decreased Vimentin and Snail, and increased Slug expression. (**B**) qRT-PCR analysis of Vimentin, E-cadherin, Snail, and Slug mRNA levels in knockdown and control cells. White, light-gray, and dark-gray bars represent the sh-NC, shIGF2BP2-1, and shIGF2BP2-2 groups, respectively; individual biological replicates are shown as open circles. E-cadherin mRNA was significantly increased in ECA109 cells (** *p* < 0.01), whereas Slug mRNA was increased in KYSE30 cells after IGF2BP2 knockdown (* *p* < 0.05). Data are shown as mean ± SD from three independent experiments.

Because IGF2BP2 knockdown reduced migration and invasion, EMT-associated markers were also examined. Western blotting showed decreased Snail expression and increased Slug expression after IGF2BP2 silencing in both ECA109 and KYSE30 cells (Figure 5A). qPCR produced similar marker-level changes (Figure 5B). These findings indicate that IGF2BP2 knockdown alters EMT-associated marker expression; however, the increased Slug expression suggests that the EMT-related changes are complex and should not be interpreted as a simple EMT-suppression pattern.

### 3.5. IGF2BP2 Is Associated with PI3K/AKT-Related Signaling in ESCC

**Figure 6:**
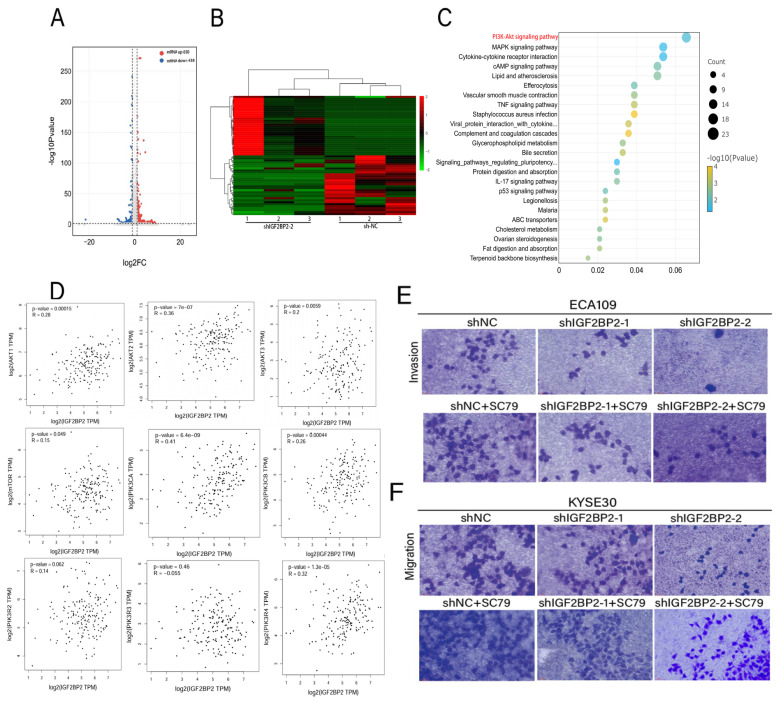
Association between IGF2BP2 and PI3K/AKT-related signaling in ESCC cells. (**A**) Volcano plot of RNA-seq data comparing ECA109 cells stably expressing shIGF2BP2-1 and sh-NC. IGF2BP2 knockdown resulted in 630 upregulated and 438 downregulated genes. (**B**) Heatmap showing differential gene expression patterns between shIGF2BP2-1 and sh-NC groups. (**C**) KEGG pathway enrichment plot showing enrichment of PI3K/AKT-related signaling, MAPK signaling, and cytokine-cytokine receptor interaction. Bubble size represents gene count, and color indicates −log10(*p* value). (**D**) GEPIA-based Pearson’s correlation analysis between IGF2BP2 and PI3K/AKT-related genes in esophageal carcinoma tissues. (**E**) Representative Transwell invasion images of ECA109 cells with or without SC79 (4 μg/mL, 24 h) after IGF2BP2 knockdown. (**F**) Representative Transwell migration images of KYSE30 cells under the same conditions. SC79 partially restored invasion or migration suppressed by IGF2BP2 depletion, supporting functional involvement of PI3K/AKT-related signaling in IGF2BP2-associated ESCC progression.

To explore downstream pathways associated with IGF2BP2, we performed RNA sequencing in ECA109 cells stably expressing shIGF2BP2-1 (SH1, SH2, and SH3) or sh-NC (NC1, NC2, and NC3). IGF2BP2 knockdown led to 438 downregulated genes and 630 upregulated genes (Figure 6A). KEGG enrichment analysis showed that these genes were enriched in PI3K/AKT-related signaling (Figure 6B,C).

We further assessed the relationship between IGF2BP2 and PI3K/AKT-related molecules using GEPIA and observed positive correlations in esophageal carcinoma tissues (Figure 6D). Western blotting showed that IGF2BP2 knockdown reduced PI3K expression in ECA109 and KYSE30 cells and decreased p-PI3K expression in KYSE30 cells, whereas p-AKT displayed an opposite trend (Figure S3). To examine the functional relevance of this pathway, we treated cells with the AKT activator SC79. SC79 partly restored the invasion and migration suppressed by IGF2BP2 knockdown, as shown by representative Transwell images and quantitative analyses (Figure 6E,F; Supplementary Figure S4). These data suggest that PI3K/AKT-related signaling may participate in IGF2BP2-associated malignant phenotypes, although they do not demonstrate direct activation of the pathway by IGF2BP2.

## 4. Discussion

Esophageal cancer is a common gastrointestinal malignancy with unsatisfactory outcomes, particularly in locally advanced disease. Radiotherapy or chemoradiotherapy combined with immune checkpoint inhibitors remains an important curative-intent approach, but many patients are diagnosed at an advanced stage, which limits treatment efficacy [25]. Therefore, identifying molecular biomarkers and therapeutic vulnerabilities remains clinically important. IGF2BP2 regulates mRNA localization, stability, and translation and has been reported to promote tumor progression in multiple cancers. For example, IGF2BP2 interacts with lncRNA HOTAIR in colon cancer [26], facilitates triple-negative breast cancer progression through EIF4A1 recruitment [27], supports T-cell acute lymphoblastic leukemia growth by stabilizing NOTCH1 [28], and contributes to melanoma progression through PD-L1 upregulation via the EGFR/STAT3 pathway.

In this study, RNA sequencing showed that IGF2BP2 was upregulated in ESCC tissues, and this finding was confirmed by IHC and qRT-PCR in independent institutional cohorts. Public database analyses also suggested that higher IGF2BP2 expression was associated with adverse clinicopathological features in ESCC. IGF2BP2 expression was higher in ESCC cell lines than in HET-1A cells, and knockdown experiments showed reduced proliferation and migration together with increased apoptosis. These results support a tumor-promoting role for IGF2BP2 in ESCC. However, because the institutional validation cohorts were relatively small and the GEPIA expression comparison was based on the TCGA-ESCA dataset, the clinical relevance of IGF2BP2 should be validated in larger ESCC-specific cohorts.

EMT contributes to invasion and metastasis by enabling epithelial tumor cells to acquire mesenchymal-like properties [29,30]. This process enhances motility and invasion and is regulated by transcription factors such as Snail1 and Snail2 [31]. In our study, IGF2BP2 knockdown changed EMT-associated markers and was accompanied by reduced migration and invasion. However, the increase in Slug after IGF2BP2 silencing was not consistent with a simple EMT-suppression model. Therefore, these findings should be regarded as EMT marker-level changes rather than proof that IGF2BP2 directly controls EMT. Additional EMT markers and mechanistic experiments are required to clarify the relationship between IGF2BP2 and EMT plasticity in ESCC.

PI3K/AKT signaling is frequently dysregulated in cancer and participates in cell survival, proliferation, angiogenesis, inflammatory signaling, and tumor microenvironment regulation [32,33]. This pathway has also been implicated in tumor progression and treatment resistance. For instance, METTL3 promotes pancreatic ductal adenocarcinoma progression and gemcitabine resistance through DDX23 mRNA m6A modification and PI3K/AKT signaling [34]. IGF2BP2 has been connected with PI3K/AKT-related pathways in several malignancies, including lung adenocarcinoma through FLT4 upregulation [35], ovarian cancer through the miR-3187-3p/ERBB4/PI3K/AKT axis [36], and head and neck squamous-cell carcinoma through PI3K/AKT signaling [37]. Moreover, PI3K/AKT signaling is associated with chemotherapy and radiotherapy resistance, and pathway inhibition may help overcome multidrug resistance [38]. YTHDC2 has also been reported to increase radioresistance through the IGF1R/AKT/S6 axis [39]. Together, these studies indicate that PI3K/AKT-related signaling may be relevant to ESCC progression and treatment response.

In line with these observations, our results suggest that PI3K/AKT-related signaling may participate in IGF2BP2-associated ESCC progression. RNA sequencing and enrichment analysis showed that genes altered after IGF2BP2 knockdown were enriched in the PI3K/AKT pathway. Western blotting showed changes in PI3K, p-PI3K, AKT, and p-AKT expression. Importantly, p-AKT increased in KYSE30 cells despite decreased p-PI3K expression after IGF2BP2 knockdown. This discrepancy may reflect cell-line-specific feedback activation or compensatory signaling, because AKT phosphorylation can be regulated by upstream inputs other than PI3K. Thus, these Western blot results should be interpreted cautiously and require further validation using p-AKT/AKT and p-PI3K/PI3K ratio analysis, pathway inhibition experiments, and additional rescue approaches. TCGA-based analysis also showed positive correlations between IGF2BP2 and PI3K/AKT-related markers in esophageal carcinoma tissues. Functionally, SC79 partially reversed the inhibitory effects of IGF2BP2 silencing on cell migration and invasion. Overall, these findings support possible involvement of PI3K/AKT-related signaling in the oncogenic effects of IGF2BP2 in ESCC but do not prove direct pathway activation by IGF2BP2.

Although our findings support a functional role for IGF2BP2 in ESCC progression, possible compensation by other IGF2BP family members should be considered. IGF2BP3, another member of the IGF2BP family, has recently been implicated in malignant progression in several cancers. In ESCC, Feng et al. reported that IGF2BP3 promoted cell migration, invasion, and epithelial-mesenchymal transition by targeting ZEB1 mRNA [40]. In addition, Chen et al. showed that IGF2BP3 functions as an m6A reader to preserve NOTCH3 mRNA stability and promote tumor metastasis in nasopharyngeal carcinoma, further supporting its role in post-transcriptional regulation and cancer progression [41]. In our transcriptomic analysis, IGF2BP3 was also expressed at a relatively high level in ESCC tumor tissues, suggesting potential functional overlap with IGF2BP2. However, IGF2BP3 was not knocked down in the present study. Therefore, we cannot exclude the possibility that IGF2BP3 contributes to, or compensates for, IGF2BP2-related malignant phenotypes. Future experiments evaluating IGF2BP3 alone and combined IGF2BP2/IGF2BP3 inhibition are needed to clarify functional redundancy within this protein family in ESCC.

From a therapeutic perspective, emerging evidence suggests that IGF2BP2 may be a druggable RNA-binding protein. Dahlem et al. identified small-molecule inhibitors of IGF2BP2/IMP2 and developed a screening strategy to interfere with IMP2-mediated oncogenic RNA regulation [42]. Feng et al. also reported the IGF2BP2 inhibitor JX5 in T-cell acute lymphoblastic leukemia, where JX5 phenocopied IGF2BP2 knockdown, reduced NOTCH1 expression, and suppressed leukemia progression in preclinical models [28]. These studies indicate that pharmacologic targeting of IGF2BP2 is feasible. Nevertheless, IGF2BP2-directed therapy remains at an early preclinical stage, and its efficacy, selectivity, and safety in ESCC have not been established. Our findings therefore provide a rationale for further investigation of IGF2BP2-targeted strategies in ESCC, but additional pharmacologic validation is required before clinical translation.

MAPK signaling was also enriched in the transcriptomic analysis, indicating that MAPK-related pathways may participate in IGF2BP2-associated ESCC progression. However, downstream MAPK components, including p-ERK, p-p38, and p-JNK, were not examined in this study. Future work should determine whether MAPK signaling cooperates with PI3K/AKT-related signaling in mediating IGF2BP2-associated oncogenic effects in ESCC.

Several limitations should be acknowledged. First, although IGF2BP2 expression was validated in institutional ESCC specimens, the patient cohorts were relatively small and available clinicopathological information was limited; therefore, the clinical relevance of IGF2BP2 requires confirmation in larger ESCC-specific cohorts. Second, our functional experiments were mainly performed in vitro, and in vivo validation was not included. Third, only selected EMT-associated markers were examined, and the EMT-related changes observed after IGF2BP2 knockdown require further mechanistic investigation. Finally, although our findings suggest that IGF2BP2 may be a candidate biomarker and potential therapeutic target, additional mechanistic, pharmacologic, in vivo, and clinical studies are needed before clinical translation.

## 5. Conclusions

In summary, IGF2BP2 was highly expressed in ESCC and was associated with clinicopathological features, supporting its potential value as a candidate biomarker and therapeutic target. Functional experiments showed that IGF2BP2 promoted malignant phenotypes in ESCC cells, including proliferation, migration, invasion, cell-cycle progression, and resistance to apoptosis. RNA-seq, Western blotting, and SC79 rescue experiments suggest that PI3K/AKT-related signaling may be involved in IGF2BP2-associated ESCC progression, but additional mechanistic validation remains necessary.

## Figures and Tables

**Table 1:** Correlation between IGF2BP2 expression and clinicopathological characteristics in ESCC patients based on TCGA data.

Characteristics		Numbers	Low Expression (*N* = 54)	High Expression (*N* = 54)	*p* Value
Age	Mean ± SD		66.0 ± 12.1	60.5 ± 11.0	0.030 *
Tumor location	Distal	82	43	39	
	Mid	23	11	12	0.098
	Proximal	3	0	3	0.989
Gender	Female	16	8	8	
	Male	92	46	46	0.677
Lymph node metastasis	No	76	41	35	
	Yes	32	13	19	0.031 *
Disease type	EAC	57	30	27	
	ESCC	51	24	27	0.023 *
Histological grade	G1	11	2	9	
	G2	41	21	20	0.015 *
	G3	31	17	14	0.014 *
	GX	25	14	11	0.169
M stage	M0	92	45	47	
	M1	2	1	1	0.349
	M1a	5	2	3	0.312
	MX	9	6	3	0.982
N stage	N0	46	21	25	
	N1	47	24	23	0.567
	N2	8	4	4	0.479
	N3	6	4	2	0.073
	NX	1	1	0	0.995
T stage	T1	21	10	11	
	T2	25	14	11	0.832
	T3	59	29	30	0.237
	T4	3	1	2	0.856
Smoking	1	30	11	19	
	2	31	11	20	0.951
	3	27	16	11	0.273
	4	20	16	4	0.006 **
Alcohol	No	35	17	18	
	Yes	73	37	36	0.533

Patients were dichotomized into low- and high-expression groups according to the median IGF2BP2 expression level. Continuous data are presented as mean ± SD, and categorical data are presented as number of patients. *p* values were calculated by Pearson’s chi-square test, except for age, which was analyzed as a continuous variable. Smoking categories 1–4 were retained according to the original TCGA clinical annotation. *p* < 0.05 was considered statistically significant; *p* < 0.01 was considered highly significant. Abbreviations: ESCC, esophageal squamous-cell carcinoma; SD, standard deviation. Bold values indicate statistical significance. * *p* < 0.05; ** *p* < 0.01.

## Data Availability

The RNA-seq data generated in this study have been deposited in the Gene Expression Omnibus (GEO) under accession number GSE336341. The records are currently private during manuscript review and are scheduled to be publicly available on 1 June 2027. A secure reviewer access token has been provided to the Editorial Office.

## Supplementary Materials

The following supporting information can be downloaded at: https://www.mdpi.com/article/10.3390/cimb48070726/s1.

## Abbreviations

ESCC, esophageal squamous-cell carcinoma; EAC, esophageal adenocarcinoma; IGF2BP2, insulin-like growth factor 2 mRNA-binding protein 2; IGF2BPs, insulin-like growth factor 2 mRNA-binding proteins; m6A, N6-Methyladenosine; TCGA, The Cancer Genome Atlas; GTEx, Genotype-Tissue Expression; GEPIA, Gene Expression Profiling Interactive Analysis; IHC, immunohistochemistry; qRT-PCR, quantitative reverse transcription polymerase chain reaction; WB, Western blotting; CCK-8, Cell Counting Kit-8; EdU, 5-ethynyl-2′-deoxyuridine; EMT, epithelial-mesenchymal transition; PI3K, phosphoinositide 3-kinase; AKT, protein kinase B; GO, Gene Ontology; KEGG, Kyoto Encyclopedia of Genes and Genomes; PBS, phosphate-buffered saline; FBS, fetal bovine serum; DAB, 3,3′-diaminobenzidine; TBST, Tris-buffered saline with Tween; PVDF, polyvinylidene difluoride; PMSF, phenylmethylsulfonyl fluoride; BCA, bicinchoninic acid; SDS-PAGE, sodium dodecyl sulfate-polyacrylamide gel electrophoresis; ECL, enhanced chemiluminescence; GEO, Gene Expression Omnibus.

## References

[1] Zhu H.C., Ma X., Ye T., Wang T.T., Wang Z.Z., Liu Q., Zhao K. (2023). Esophageal cancer in China: Practice and research in the new era. Int. J. Cancer.

[2] Rogers J.E., Sewastjanow-Silva M., Waters R.E., Ajani J.A. (2022). Esophageal cancer: Emerging therapeutics. Expert Opin. Ther. Targets.

[3] Yang H., Wang F., Hallemeier C.L., Lerut T., Fu J.H. (2024). Oesophageal Cancer. Lancet.

[4] The Cancer Genome Atlas Research Network (2017). Integrated genomic characterization of oesophageal carcinoma. Nature.

[5] Liang H., Fan J.H., Qiao Y.L. (2017). Epidemiology, etiology, and prevention of esophageal squamous cell carcinoma in China. Cancer Biol. Med..

[6] Zhao J., He Y.T., Zheng R.S., Zhang S.W., Chen W.Q. (2012). Analysis of esophageal cancer time trends in China, 1989-2008. Asian Pac. J. Cancer Prev..

[7] Shapiro J., van Lanschot J.J.B., Hulshof M.C.C.M., van Hagen P., van Berge Henegouwen M.I., Wijnhoven B.P.L., van Laarhoven H.W.M., Nieuwenhuijzen G.A.P., Hospers G.A.P., Bonenkamp J.J. (2015). Neoadjuvant chemoradiotherapy plus surgery versus surgery alone for oesophageal or junctional cancer (CROSS): Long-term results of a randomised controlled trial. Lancet Oncol..

[8] Bell J.L., Wächter K., Mühleck B., Pazaitis N., Köhn M., Lederer M., Hüttelmaier S. (2013). Insulin-like growth factor 2 mRNA-binding proteins (IGF2BPs): Post-transcriptional drivers of cancer progression?. Cell. Mol. Life Sci..

[9] Christiansen J., Kolte A.M., Hansen T., Nielsen F.C. (2009). IGF2 mRNA-binding protein 2: Biological function and putative role in type 2 diabetes. J. Mol. Endocrinol..

[10] Cao J., Mu Q., Huang H. (2018). The roles of insulin-like growth factor 2 mRNA-binding protein 2 in cancer and cancer stem cells. Stem Cells Int..

[11] Dai N. (2020). The diverse functions of IMP2/IGF2BP2 in metabolism. Trends Endocrinol. Metab..

[12] Huang H., Weng H., Sun W.J., Qin X., Shi H.L., Wu H.Z., Zhao B.S., Mesquita A., Liu C., Yuan C.L. (2018). Recognition of RNA N6-methyladenosine by IGF2BP Proteins Enhances mRNA Stability and Translation. Nat. Cell Biol..

[13] Wu Y., Zhou C., Yuan Q. (2018). Role of DNA and RNA N6-Adenine methylation in regulating stem cell fate. Curr. Stem Cell Res. Ther..

[14] Li T., Forbes M.E., Fuller G.N., Li J.B., Yang X.J., Zhang W. (2020). IGFBP2: Integrative hub of developmental and oncogenic signaling network. Oncogene.

[15] Wang H., Rosen D.G., Wang H., Fuller G.N., Zhang W., Liu J. (2006). Insulin-like growth factor-binding protein 2 and 5 are differentially regulated in ovarian cancer of different histologic types. Mod. Pathol..

[16] So A.I., Levitt R.J., Eigl B., Fazli L., Muramaki M., Leung S., Cheang M.C., Nielsen T.O., Gleave M., Pollak M. (2008). Insulin-like growth factor binding protein-2 is a novel therapeutic target associated with breast cancer. Clin. Cancer Res..

[17] Migita T., Narita T., Asaka R., Miyagi E., Nagano H., Nomura K., Matsuura M., Satoh Y., Okumura S., Nakagawa K. (2010). Role of insulin-like growth factor binding protein 2 in lung adenocarcinoma: IGF-independent antiapoptotic effect via caspase-3. Am. J. Pathol..

[18] Kim Y.W., Bae S.M., Kim Y.W., Park D.C., Lee K.H., Liu H.B., Kim I.-W., Jang C.K., Ahn W.S. (2013). Target-based molecular signature characteristics of cervical adenocarcinoma and squamous cell carcinoma. Int. J. Oncol..

[19] Ben-Shmuel A., Shvab A., Gavert N., Brabletz T., Ben-Ze’ev A. (2013). Global analysis of L1-transcriptomes identified IGFBP-2 as a target of ezrin and NF-κB signaling that promotes colon cancer progression. Oncogene.

[20] Matuschek C., Rudoy M., Peiper M., Gerber P.A., Hoff N.P., Buhren B.A., Flehmig B., Budach W., Knoefel W.T., Bojar H. (2011). Do insulin-like growth factor associated proteins qualify as a tumor marker? Results of a prospective study in 163 cancer patients. Eur. J. Med. Res..

[21] Chua C.Y., Liu Y., Granberg K.J., Hu L., Haapasalo H., Annala M.J., Cogdell D.E., Verploegen M., Moore L.M., Fuller G.N. (2016). IGFBP2 potentiates nuclear EGFR-STAT3 signaling. Oncogene.

[22] Li Y., Xiao Z., Wang Y., Zhang D., Chen Z. (2024). The m6A reader IGF2BP2 promotes esophageal cell carcinoma progression by enhancing EIF4A1 translation. Cancer Cell Int..

[23] Zhao R., Li T., Zhao X., Yang Z., Ma L., Wang X. (2024). The m6A reader IGF2BP2 promotes the progression of esophageal squamous cell carcinoma cells by increasing the stability of OCT4 mRNA. Biochem. Cell Biol..

[24] Jia Y., Liu S., Zhang M., Wu X., Chen X., Xing M., Hou X., Jiang W. (2024). The m6A reader IGF2BP2 promotes ESCC progression by stabilizing HDGF mRNA. J. Cancer Res. Ther..

[25] Wu J., Deng R., Ni T.T., Zhong Q., Tang F., Li Y., Zhang Y. (2022). Efficacy and safety of radiotherapy/chemoradiotherapy combined with immune checkpoint inhibitors for locally advanced stages of esophageal cancer: A systematic review and meta-analysis. Front. Oncol..

[26] Qu X., Alsager S., Zhuo Y., Shan B. (2019). HOX transcript antisense RNA (HOTAIR) in cancer. Cancer Lett..

[27] Xia T., Dai X.Y., Sang M.Y., Zhang X., Xu F., Wu J., Shi L., Wei J., Ding Q. (2023). IGF2BP2 Drives Cell Cycle Progression in Triple-Negative Breast Cancer by Recruiting EIF4A1 to Promote the m6A-Modified CDK6 Translation Initiation Process. Adv. Sci..

[28] Feng P., Chen D., Wang X., Li Y., Li Z., Li B., Zhang Y., Li W., Zhang J., Ye J. (2022). Inhibition of the m6A reader IGF2BP2 as a strategy against T-cell acute lymphoblastic leukemia. Leukemia.

[29] Huang Y.H., Hong W.Q., Wei X.W. (2022). The molecular mechanisms and therapeutic strategies of EMT in tumor progression and metastasis. J. Hematol. Oncol..

[30] Nieto M.A., Huang R.Y., Jackson R.A., Thiery J.P. (2016). EMT: 2016. Cell.

[31] Nieto M.A. (2013). Epithelial Plasticity: A Common Theme in Embryonic and Cancer Cells. Science.

[32] Hoxhaj G., Manning B.D. (2020). The PI3K-AKT network at the interface of oncogenic signalling and cancer metabolism. Nat. Rev. Cancer.

[33] Yu L., Wei J., Liu P. (2022). Attacking the PI3K/Akt/mTOR signaling pathway for targeted therapeutic treatment in human cancer. Semin. Cancer Biol..

[34] Lin C.J., Li T., Wang Y., Lai S.H., Huang Y., Guo Z.Y., Zhang X., Weng S. (2023). METTL3 enhances pancreatic ductal adenocarcinoma progression and gemcitabine resistance through modifying DDX23 mRNA N6 adenosine methylation. Cell Death Dis..

[35] Fang H., Sun Q., Zhou J., Zhang H.J., Song Q., Zhang H., Yu G., Guo Y., Huang C., Mou Y. (2023). M6A methylation reader IGF2BP2 activates endothelial cells to promote angiogenesis and metastasis of lung adenocarcinoma. Mol. Cancer.

[36] Wang S.T., Li Z.H., Zhu G.H., Lan H., Hu C.H., Wang K., Cui K., Hao C. (2021). RNA-binding protein IGF2BP2 enhances circ_0000745 abundancy and promotes aggressiveness and stemness of ovarian cancer cells via the microRNA-3187-3p/ERBB4/PI3K/AKT axis. J. Ovarian Res..

[37] Yu D., Pan M., Li Y.S., Lu T., Wang Z.H., Liu C., Hu G. (2022). RNA N6-methyladenosine reader IGF2BP2 promotes lymphatic metastasis and epithelial-mesenchymal transition of head and neck squamous carcinoma cells via stabilizing slug mRNA in an m6A-dependent manner. J. Exp. Clin. Cancer Res..

[38] He Y., Sun M.M., Zhang G.G., Yang J., Chen K.S., Xv W.W., Li B. (2021). Targeting PI3K/Akt signal transduction for cancer therapy. Signal Transduct. Target. Ther..

[39] He J.J., Li Z., Rong Z.X., Gao J., Mu Y., Guan Y.D., Ren X.-X., Zi Y.-Y., Liu L.-Y., Fan Q. (2020). m6A Reader YTHDC2 Promotes Radiotherapy Resistance of Nasopharyngeal Carcinoma via Activating IGF1R/AKT/S6 Signaling Axis. Front. Oncol..

[40] Feng Y., Lin Y., Jiang Z., Wu L., Zhang Y., Wu H., Yuan X. (2023). Insulin-like growth factor-2 mRNA-binding protein 3 promotes cell migration, invasion, and epithelial–mesenchymal transition of esophageal squamous cell carcinoma cells by targeting zinc finger E-box-binding homeobox 1 mRNA. Mol. Carcinog..

[41] Chen B., Huang R., Xia T., Wang C., Xiao X., Lu S., Chen X., Ouyang Y., Deng X., Miao J. (2023). The m6A reader IGF2BP3 preserves NOTCH3 mRNA stability to sustain Notch3 signaling and promote tumor metastasis in nasopharyngeal carcinoma. Oncogene.

[42] Dahlem C., Abuhaliema A., Kessler S.M., Kröhler T., Zoller B.G.E., Chanda S., Wu Y., Both S., Müller F., Lepikhov K. (2022). First Small-Molecule Inhibitors Targeting the RNA-Binding Protein IGF2BP2/IMP2 for Cancer Therapy. ACS Chem. Biol..

