# Supplementary material for: IGF2BP2 Promotes Esophageal Squamous-Cell Carcinoma Progression with Potential Involvement of PI3K/AKT Signaling
Source: Curr Issues Mol Biol. 2026 Jul 16;48(7):726. doi: 10.3390/cimb48070726 (PMC13406996; doi:10.3390/cimb48070726)
Supplement: Supplementary file 1 [file cimb-48-00726-s001.zip › cimb-4410519-supplementary.pdf]

Supplementary Table S1. Target sequences of shRNA constructs used for IGF2BP2 knockdown.

	**Target sequence：**
shIGF2BP2-1	GGTGCCTGCAGCGGTAATATA
shIGF2BP2-2	GTTGGCCCAGGGCGTTAAATT
shIGF2BP2-3	AGTGAAGCTGGAAGCGCATAT
Negative control	GTTCTCCGAACGTGTCACGTT

Three independent shRNA sequences targeting human *IGF2BP2* (shIGF2BP2-1, shIGF2BP2-2, and shIGF2BP2-3) and a non-targeting scramble sequence (negative control) were designed and synthesized. All constructs were cloned into the lentiviral vector pPLK/GFP+Puro for stable transduction in ESCC cell lines.

Supplementary Table S2. Primer sequences used for qRT-PCR analysis.

Gene	Forward	Reverse
IGF2BP2	CATCGGAAAGGAGGGCTTGA	ATGGTGACAGGCTTCTCTGC
hGAPDH	TTCGTCATGGGTGTGAACCA	GTCTTCTGGGTGGCAGTGAT
Vimentin	GGACCAGCTAACCAACGACA	AAGGTCAAGACGTGCCAGAG
E-cadherin	TGGAACAGGGACACTTCTGC	CCCCGTGTGTTAGTTCTGCT
snail	CCCCAATCGGAAGCCTAACT	AGATGAGCATTGGCAGCGA
slug	AGCATACAGCCCCATCACTG	CTCGCCCCAAAGATGAGGAG

Forward and reverse primer sequences for IGF2BP2, GAPDH (internal reference), and EMT-associated markers (Vimentin, E-cadherin, Snail, and Slug) used in quantitative reverse transcription PCR (qRT-PCR) experiments. All primers were designed to span exon－exon junctions to avoid genomic DNA amplification. GAPDH was used as the housekeeping gene for normalization of relative gene expression.

Supplementary Table S3. Demographic and clinicopathological characteristics of patients included in the qPCR validation cohort

**Characteristic**	**qPCR validation cohort (n = 16)**
Age, years, median (range)	63.5 (49-73)
Age category, years	
<60	7 (43.8)
≥60	9 (56.2)
Sex	
Male	11 (68.8)
Female	5 (31.2)
Smoking status	
Yes	3 (18.8)
No	13 (81.2)
Alcohol consumption	
Yes	1 (6.2)
No	15 (93.8)
Tumor location	
Upper thoracic esophagus	2 (12.5)
Middle thoracic esophagus	3 (18.8)
Lower thoracic esophagus	11 (68.8)
Histological grade	
G1, well differentiated	0 (0.0)
G2, moderately differentiated	4 (25.0)
G3, poorly differentiated	8 (50.0)
Unknown	4 (25.0)
T stage	

T1	0 (0.0)
T2	4 (25.0)
T3	7 (43.8)
T4	5 (31.2)
N stage	
N0	0 (0.0)
N1	1 (6.2)
N2	11 (68.8)
N3	4 (25.0)
M stage	
M0	16 (100.0)
M1	0 (0.0)
Distant metastasis	
Yes	0 (0.0)
No	16 (100.0)

Data are presented as n (%) unless otherwise indicated. The qPCR validation cohort was independent of the specimens used for immunohistochemistry. All specimens were residual endoscopic biopsy specimens obtained for routine pathological diagnosis. ESCC, esophageal squamous cell carcinoma; qPCR, quantitative polymerase chain reaction.

Supplementary Table S4. Antibodies used for western blotting

Primary antibodies

Target	Vendor	Catalog No.	Dilution	Application	Primary antibody incubation
IGF2BP2	Proteintech	11601-1-AP	1:2000	WB	4°C overnight
PI3K	CST	4257S	1:1000	WB	4°C overnight

Target	Vendor	Catalog No.	Dilution	Application	Primary antibody incubation
p-PI3K	CST	4228S	1:1000	WB	4°C overnight
AKT	CST	9272S	1:1000	WB	4°C overnight
p-AKT	CST	4060S	1:2000	WB	4°C overnight
E-cadherin	CST	3195T	1:1000	WB	4°C overnight
Vimentin	CST	5741T	1:1000	WB	4°C overnight
Snail	CST	3879T	1:1000	WB	4°C overnight
Slug	CST	9585T	1:2000	WB	4°C overnight
GAPDH	Proteintech	60004-1-Ig	1:50000	WB	4°C overnight

Secondary antibodies

Secondary antibody	Vendor	Catalog No.	Dilution	Application	Incubation
Goat anti-rabbit IgG	Proteintech	SA00001-2	1:10000	WB	Room temperature, 1 h
Goat anti-mouse IgG	Proteintech	SA00001-1	1:10000	WB	Room temperature, 1 h

Note: WB, western blotting.

Supplementary Figure S1. Validation of IGF2BP2 knockdown efficiency in ESCC cell lines following lentiviral shRNA transduction.

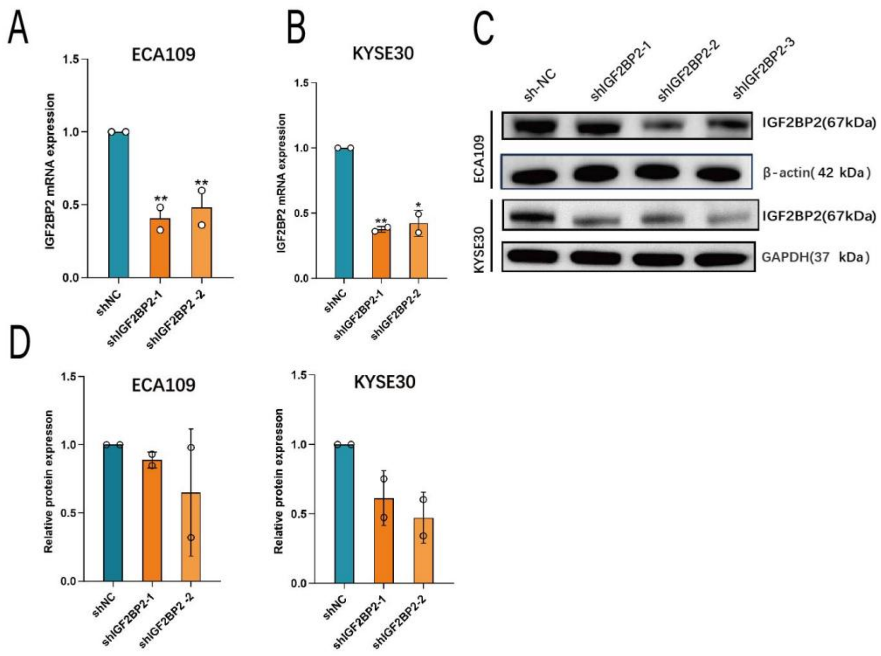

(A, B) Relative IGF2BP2 mRNA expression levels in ECA109 (A) and KYSE30 (B) cells stably transduced with scramble shRNA (shNC), shIGF2BP2-1, shIGF2BP2-2, or shIGF2BP2-3, as determined by qRT-PCR. GAPDH was used as the internal reference. Data are presented as mean ± SD from three independent experiments. ***P < 0.001 versus shNC group. (C, D) Protein expression of IGF2BP2 in ECA109 (C) and KYSE30 (D) cells after stable shRNA-mediated knockdown, as assessed by western blotting. GAPDH served as the loading control. All three shRNA constructs achieved effective knockdown at both mRNA and protein levels.

Supplementary Figure S2. IGF2BP2 knockdown suppresses ESCC cell proliferative activity as assessed by EdU incorporation assay.

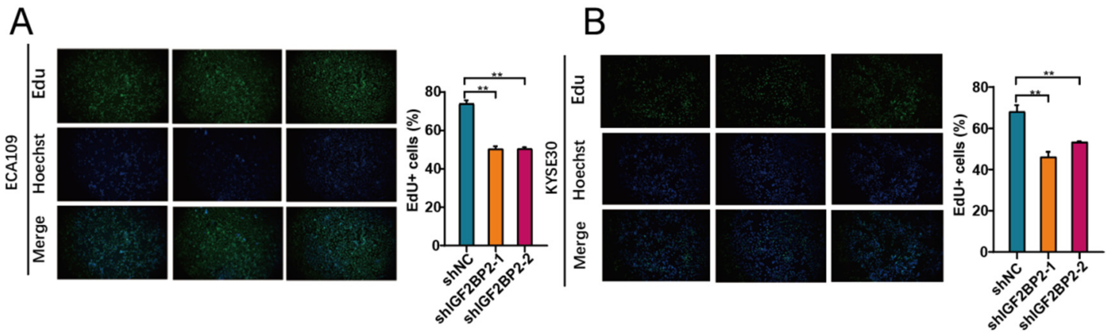

(A, B) Representative fluorescence microscopy images (200×) and corresponding quantification of EdU-positive cells in ECA109 (A) and KYSE30 (B) cell lines following stable knockdown of IGF2BP2 (shIGF2BP2-1, shIGF2BP2-2, shIGF2BP2-3) compared with the scramble control (shNC). EdU-incorporating cells (red) represent actively proliferating cells; nuclei were counterstained with Hoechst 33342 (blue). The proportion of EdU-positive cells was significantly reduced upon IGF2BP2 depletion, consistent with impaired DNA synthesis and reduced cell proliferative capacity. Data are presented as mean ± SD from three independent experiments. *$P < 0.05$, **$P < 0.01$, ***$P < 0.001$ versus shNC group.

Supplementary Figure S3. Effects of IGF2BP2 knockdown on the expression of PI3K/AKT signaling pathway proteins and EMT-associated markers in ESCC cell lines.

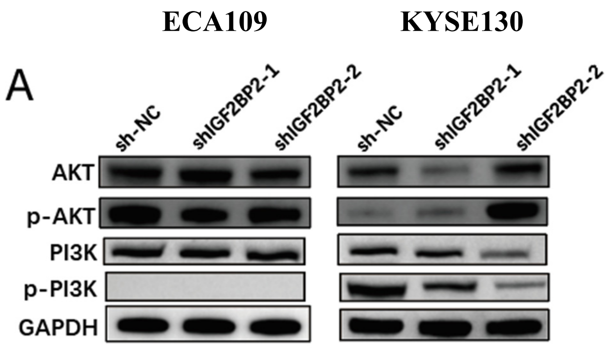

(A) Representative Western blot images showing the protein expression levels of AKT, phosphorylated AKT (p-AKT), PI3K, and phosphorylated PI3K (p-PI3K) in ECA109 and KYSE30 cells stably transduced with scramble shRNA (sh-NC), shIGF2BP2-1, or shIGF2BP2-2. GAPDH was used as the loading control. These representative results suggest that IGF2BP2 knockdown may affect PI3K/AKT pathway–related protein expression in ESCC cells.

Supplementary Figure S4. Quantitative analysis of Transwell invasion and migration assays after SC79 treatment.

A **B**

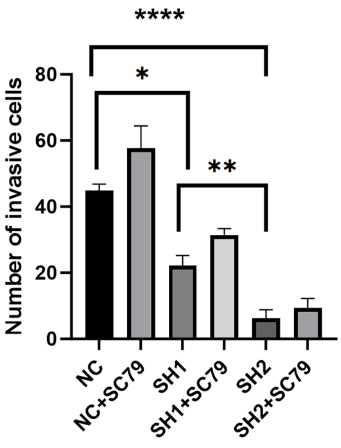

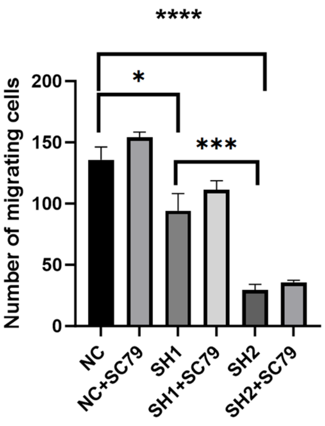

C **D**

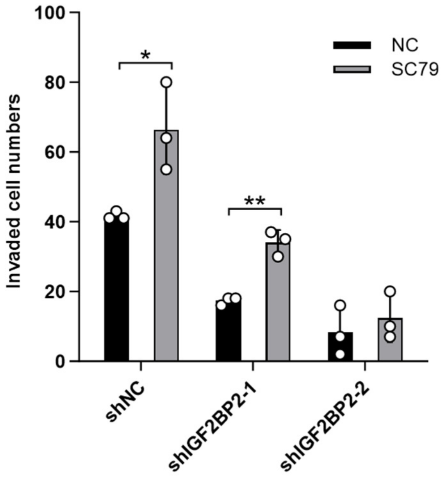

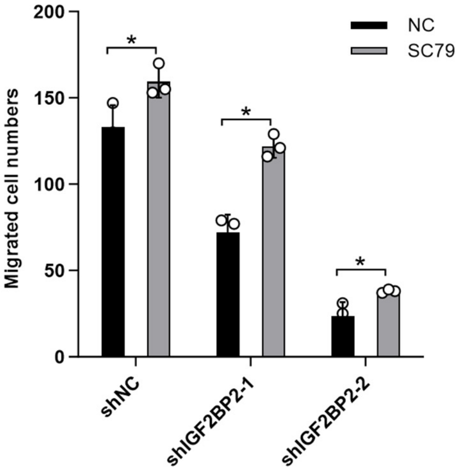

(A) Quantification of invaded ECA109 cells after IGF2BP2 knockdown with or without treatment with the AKT activator SC79. (B) Quantification of migrated ECA109 cells after IGF2BP2 knockdown with or without SC79 treatment. (C) Quantification of invaded KYSE30 cells after IGF2BP2 knockdown with or without SC79 treatment. (D) Quantification of migrated KYSE30 cells after IGF2BP2 knockdown with or without SC79 treatment. Cells were transfected with shNC, shIGF2BP2-1, or shIGF2BP2-2 and treated with SC79 where indicated. Invaded or migrated cells were counted from microscopic fields. Data are presented as the mean ± SD. Statistical significance was determined using one-way ANOVA. $P < 0.05$; $P < 0.01$; $P < 0.001$; $P < 0.0001$.
